# Meta-analysis of diagnostic and prognostic value of miR-126 in non-small cell lung cancer

**DOI:** 10.1042/BSR20200349

**Published:** 2020-05-11

**Authors:** Lin Sun, Hongbin Zhou, Ying Yang, Jianguo Chen, Yong Wang, Min She, Chang Li

**Affiliations:** Geriatric Pulmonary Department, Chongzhou People’s Hospital, Chengdu 611230, Sichuan, China

**Keywords:** diagnosis, Meta-analysis, miR-126, NSCLC, prognosis

## Abstract

In recent years, many studies on the relationship between the expression of microRNA-126 (miR-126) and the diagnostic and prognostic value of non-small cell lung cancer (NSCLC) have been made, but the results were still controversial. The aim is to explore the expression of miR-126 and the diagnosis and prognosis value of NSCLC, and to provide relevant evidence for clinical diagnosis and treatment. Literature related to miR-126 and NSCLC were searched in PubMed, Embase, Cochrane Library, Web of Science, CNKI, and Wanfang from the inception to February 2020. Stata 15.0 was used for meta-analysis. The diagnostic value data were used to calculate the pooled sensitivity, specificity, diagnostic odds ratio (DOR), positive likelihood ratio (PLR), negative likelihood ratio (NLR), and the prognostic value data were used to calculate the pooled risk ratio (hazard ratio, HR) of overall survival (OS) and its 95% confidence interval (95% CI). Thirteen studies were included, among which five were related to diagnosis containing 439 patients and 463 healthy controls, and eight related to prognosis containing 1102 patients. The results of miR-126 expression and diagnostic value of NSCLC showed that the pooled sensitivity was 0.83 (95% CI: 0.59–0.94), specificity = 0.83 (95% CI: 0.71–0.90), PLR = 4.78 (95% CI: 2.97–7.69), NLR = 0.20 (95% CI: 0.08–0.54), DOR = 23.48 (95% CI: 7.87–70.10), and the area under the summ ary receiver operating characteristic curve (SROC) was 0.89 (95% CI: 0.86–0.91). The results of prognostic value indicated that the expression of miR-126 was related to the OS of NSCLC (HR = 0.79, 95% CI: 0.63–0.98). In conclusion, the expression of miR-126 has medium diagnostic value, and it is related to the prognosis of patients with NSCLC, with poor prognosis of miR-126 low expression.

## Introduction

Lung cancer is one of the leading causes of cancer deaths in the world, accounting for approximately one-fifth of all cancer deaths [1], including two major groups: non-small cell lung cancer (NSCLC), is approximately 85% of cases and small cell lung cancer (SCLC) is approximately 15%. The most common subtypes of NSCLC are adenocarcinoma and squamous cell lung cancer. The prognosis of lung cancer depends on whether early clinical diagnosis and treatment are conducted. Surgical resection is the most effective treatment for now but the recurrence rate after having the surgery for 5 years is 30% [2]. Radiotherapy is also one of the optional treatments for patients with an unresectable area in early stage of lung cancer [3]. Unfortunately, only 16% of patients present with localized diseases at the time of initial diagnosis, and the vast majority of patients are diagnosed with regional (22%) or distant (57%) metastasis and lose the opportunity for surgery. The 5-year survival rate ranges from 67% in T1N0 patients to 23% in T1-3N2 patients to approximately 1–10% in metastatic patients [4]. Many patients are at an advanced stage when they are diagnosed and lose the opportunity for early treatment. Since the NSCLC patient accounts for the majority of lung cancer patient, it is of great clinical significance to look for NSCLC markers with high sensitivity and specificity. MicroRNAs (miRNAs) are a class of regulatory noncoding RNAs with a length of 20–25 bases, which can not only recognize target mRNA, degrade target mRNA, or inhibit target mRNA translation by complementary base pairing [5], but also interact with proto-oncogenes and tumor suppressor factors of cells to participate in the expession of oncogene [6,7]. Studies have found that more than 50% of the genes are located in cancer-related genomic regions or fragile sites [8], suggesting that miRNAs may play an important role in the formation and development of some tumors. As an important member of the miRNAs, microRNA-126 (miR-126) is located in intron 7 of the epidermal growth factor-like protein 7 gene [9] and participates in a wide range of biological function expression. Studies have shown that miR-126 can regulate the induction and function of CD4^+^Foxp3^+^ regulatory T cells through the PI3K/AKT pathway [10]. It is worth noting that miR-126 also plays a crucial role in the progression of NSCLC, for example, the expression level of miR-126 is significantly down-regulated in NSCLC [11–13]. Studies by Tafsiri et al. [12] show that there is a correlation between miR-126 and TNM staging of NSCLC. This indicates that miR-126 can be used as a biomarker for the early diagnosis of NSCLC. Morever, miR-126 can regulate the biological characteristics of NSCLC through different mechanisms. For example, miR-126 can regulate the proliferation of NSCLC through Stat3 [14]. In addition, the decreased expression of miR-126 may enhance the adhesion, migration, and invasion of NSCLC cells by increasing Crk protein [15]. These results suggest that miR-126 may be an important regulatory gene in the genesis and development of NSCLC. Several studies have shown that in NSCLC patients, patients with high expression of miR-126 had a better prognosis than patients with low expression [16,30]. Therefore, this suggests that miR-126 could be used as a prognostic biomarker of NSCLC.

So far, many researchers have published data on the diagnostic and prognostic value of miR-126 in lung cancer, which have raised concerns about the effectiveness of miR-126 as a biomarker. In the present study, we conducted a meta-analysis on these published studies to estimate the diagnostic and prognostic value of miR-126 in lung cancer.

## Materials and methods

### Retrieval strategy

The related literature databases PubMed, Embase, Cochrane Library, and Web of Science and Chinese database (CNKI and Wanfang database) were searched to evaluate the diagnostic value of miR-126 in NSCLC. Research was selected from the inception to February 2020, with ‘microRNA-126’, ‘miRNA-126’, ‘microRNA126’, ‘miR-126’, ‘miRNA126’, ‘miR-126-3p’, ‘lung tumor’, ‘NSCLC’, ‘non-small cell lung cancer’, ‘lung neoplasms’ and ‘lung cancer’ as keywords. The comprehensive database search was carried out independently by two authors.

### Literature selection criteria

#### Inclusion criteria

(1) miR-126 in NSCLC was studied. (2) The samples included in the diagnostic value were plasma, serum or secretions, while tissue, plasma, and serum were included in the prognostic value. (3) The relationship between miR-126 and the result of overall survival (OS) or diagnostic accuracy was studied.

#### Exclusion criteria

(1) Studies on miR-126 expression and prognosis were investigated, but no survival analysis was performed. (2) To study a group of miRNAs rather than a single miR-126. (3) Letters, case reports, reviews, conference summaries, animal or laboratory studies. (4) Lack of important information such as risk ratio (hazard ratio, HR), 95% confidence interval (95% CI) and *P*-value, or diagnostic sensitivity and specificity cannot be extracted.

### Quality evaluation

Quality assessment of diagnostic accuracy studies (QUADAS) was used to study the diagnostic value [17]. The QUADAS criteria includs 14 evaluation items for systematic review of diagnostic accuracy studies. Each of the 14 items was rated as yes (score 1), no (score –1), or unclear (score 0). When the QUADAS score was 11, the quality of the study is defined as high quality. Any differences between the two researchers were resolved through discussion or the assistance of third researcher.

The Newcastle–Ottawa scale (NOS) was used to assess the methodological quality of prognostic value studies [18], and three components (selection, comparability, exposure) and eight items were evaluated. In the selection and exposure part, a quality research project gets 1 star, while a comparable category can only get up to 2 stars. The range of quality evaluation value is 0–9 stars. Those awarded less than 6 are low-quality studies. In general, studies awarded at least 6 are considered to be included in the meta-analysis.

### Data extraction

The study of diagnostic value extracted the following information: (1) first author, year of publication, tumor grade, detection method, and cut-off value; (2) extract data by designing a form which includes sensitivity, specificity, true positive number, false positive number, false negative number, true negative number, etc.

The prognostic value of the study extracted the following information: (1) first author, year of publication, tumor grade, total number of samples, determination method, and cut-off value; (2) HR and its 95% CI.

### Statistical analysis

Stata 15.0 software was used to analyze the data. The inconsistency index (*I^2^*) and its test *P*-value were used to evaluate the heterogeneity between studies. The bivariate mixed effect regression model was used to analyze the pooled diagnostic indicators. In the study of diagnostic value, the diagnostic threshold effect was evaluated by receiver operating curve (ROC) and the Spearman correlation coefficient between sensitivity and specificity. The typical shoulder-arm representation in ROC space and the strong positive correlation between the logarithm of sensitivity and the logarithm of 1-specificity would indicate the existence of threshold effect. The total statistics and their forest plots of sensitivity, specificity, positive likelihood ratio (PLR), negative likelihood ratio (NLR) and diagnostic odds ratio (DOR) were calculated with corresponding 95% CI. The ROC (summary receiver operating characteristic curve, SROC) of the area under the curve (AUC) was obtained. AUC values ranged from 0.5 to 1.0, if it was close to 0.5, the diagnostic performance is poor; if it was close to 1.0, the diagnostic performance is good. Deeks’s funnel plot was used to evaluate publication bias. In the study of prognostic value, the combined HR and 95% CI were calculated. If there was heterogeneity, subgroups analysis of ethnicity and sample sources were performed to detect the sources of heterogeneity. Sensitivity analysis was carried out to analyze the robustness of the results. Funnel plot and Egger’s test were used to evaluate publication bias. If *P*<0.05, it showed that the difference was statistically significant.

## Results

### Literature research and characteristic of studies

**Figure 1:**
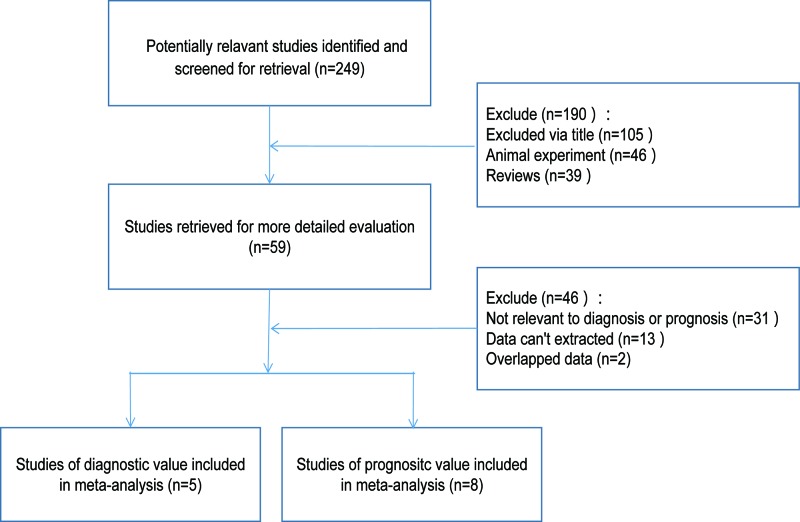
A flow diagram of the study selection process

**Figure 2:**
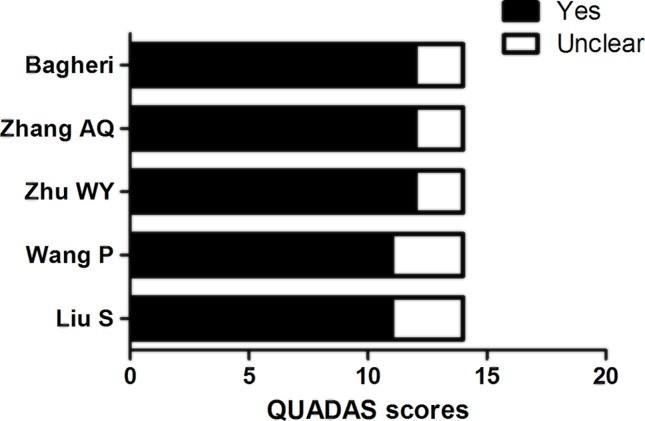
Bar chart of quality score of diagnostic test literature

Initially, 249 articles were retrieved by the keywords. Second, through reviewing the titles and abstracts of all articles, 190 was excluded. Third through checking the full-text and data integrity, 46 articles were further excluded. Finally, 13 studies that met all the inclusion criteria were included in the present study, containing 5 studies on the diagnostic accuracy of miR-126 and NSCLC [19–23] and 8 studies on miR-126 and the prognosis of NSCLC [16,24–30]. Specific screening flow chart was presented in Figure 1. The basic characteristics and method of the included literature related to the accuracy of diagnosis is shown in Table 1 and the basic characteristics and method of the included literature related to the prognosis was shown in Table 2. The quality bar chart of the literature on diagnostic tests was shown in Figure 2. Therefore, the quality score of the literature included in the diagnostic value was 11 or more, and that of the prognostic value was 6 or more.

**Table 1:** Characters of included studies of diagnostic value

First author	Year	Patients (control)	Country	Specimen	Tumor stage	Cutoff	Normalizers	Measurements	TP	FP	FN	TN	QUADAS scores
Liu et al. [19]	2011	130 (170)	China	Plasma	I–IIIA	NR	has-miR-126	qRT-PCR	60	17	70	153	11
Wang et al. [20]	2015	94 (111)	China	Serum	IA–IIB	NR	cel-miR-39	qRT-PCR	78	41	16	70	11
Zhu et al. [21]	2016	112 (40)	China	Serum	0–IIIA	0.9931	U6 snRNA	qRT-PCR	68	3	44	37	12
Shang [22]	2017	127 (112)	China	Serum	I–IV	0.95	miR-16	qRT-PCR	122	19	5	93	12
Bagheri et al. [23]	2018	30 (30)	Iran	Sputum	I–IV	NR	U6 snRNA	qRT-PCR	29	7	1	23	12

Abbreviations: FN, false negative; FP, false positive; NR, non-report; qRT-PCR, quantitative real-time polymerase chain reaction; TN, true negative; TP, true positive.

**Table 2:** Characters of included studies of prognostic value

First author	Year	Country	Patients	Specimen	Tumor stage	miR-126 assay	Cut-off value	HR (95% CI)	End point	Follow-up (median or mean month)	NOS score
Yang et al. [24]	2012	China	442	Tissue	I–IV	qRT-PCR	Median	0.782 (0.65–0.95)	OS	24.39–29.28	8
Kim et al. [16]	2014	Korea	72	Tissue	I–IV	qRT-PCR	Median	0.44 (0.16–1.2)	OS	31	7
Li et al. [28]	2014	China	49	Tissue	NR	qRT-PCR	Median	0.71 (0.13–3.94)	OS	39	7
Chen et al. [26]	2015	China	113	Tissue	I–III	qRT-PCR	Median	0.48 (0.29–0.8)	OS	NR	6
Begum et al. [25]	2015	U.S.A.	114	Tissue	I–IV	qRT-PCR	Median	0.76 (0.43–1.30)	OS	46.3	8
Xu et al. [27]	2017	China	196	Plasma	I–III	qRT-PCR	Median	1.32 (0.93–1.87)	OS	56.7	8
Switlik et al. [29]	2017	Poland	33	Tissue	I–III	qRT-PCR	Median	0.93 (0.76–1.13)	OS	NR	7
Ulivi et al. [30]	2019	Italy	83	Serum	I–IIIA	qRT-PCR	Median	0.62 (0.43–0.90)	OS	80	8

Abbreviation: qRT-PCR, quantitative real-time polymerase chain reaction.

### Meta-analysis results

**Figure 3:**
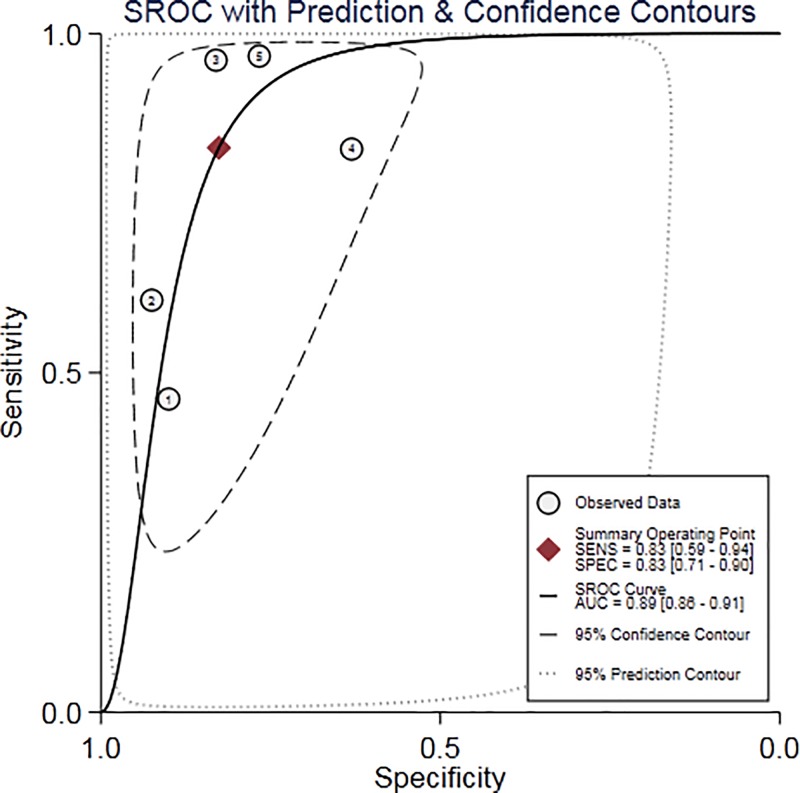
SROC curve for the accuracy of miR-126 in the diagnosis of NSCLC Abbreviation: SENS, sensitivity; SPEC, specificity.

**Figure 4:**
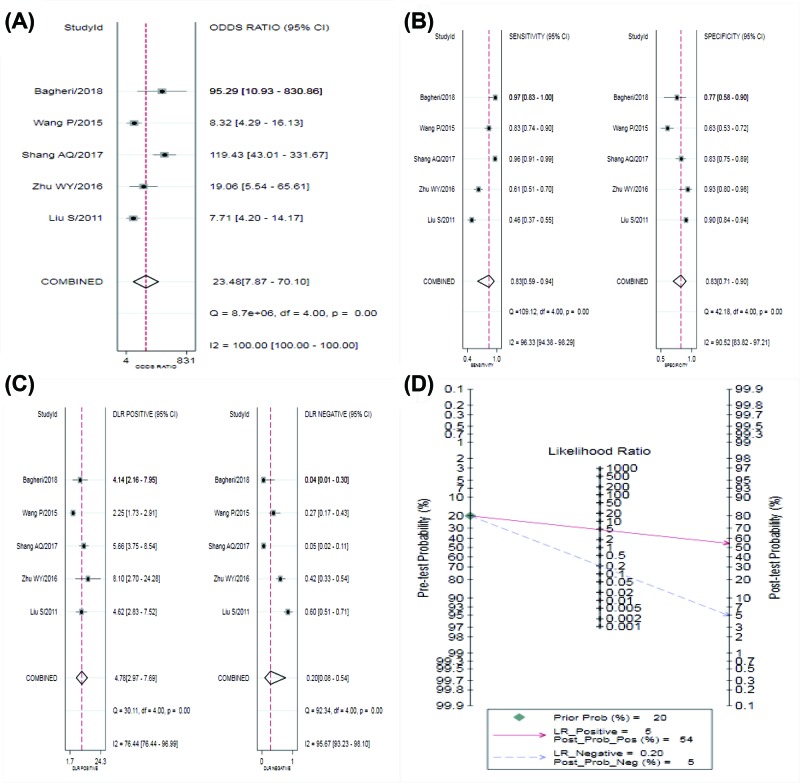
Forest plot of miR-126 for the diagnosis of NSCLC (**A**) DOR; (**B**) sensitivity and specificity; (**C**) PLR and NLR; (**D**) Fagan’s Nomogram.

**Figure 5:**
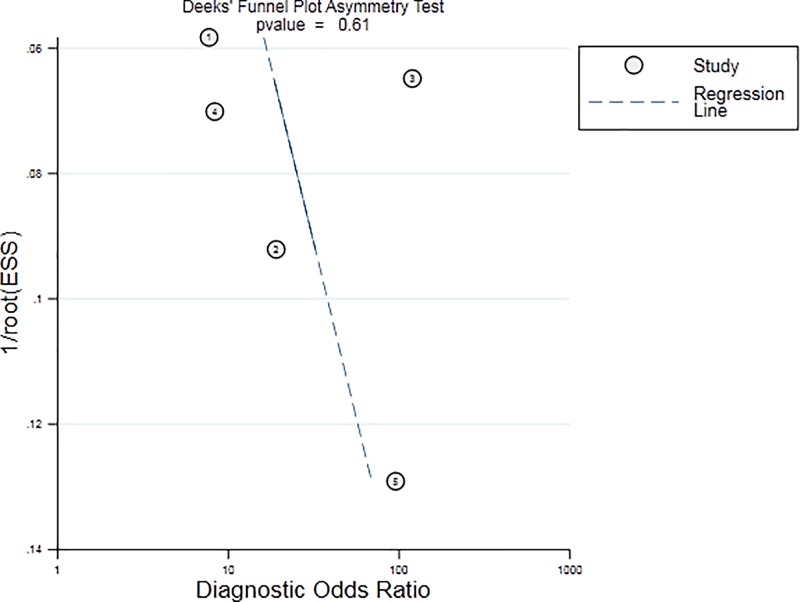
Funnel plot of miR-126 for the diagnosis of NSCLC

According to the results of diagnostic accuracy analysis, significant heterogeneity is found in the studies of sensitivity (*P*=0.00, *I^2^* = 96.33), specificity (*P*=0.00, *I^2^* = 90.52%), PLR (*P*=0.00, *I^2^* = 76.44%), NLR (*P*=0.00, *I^2^* = 95.67%), and DOR (*P*=0.00, *I^2^* = 100.0%). There was no significant threshold effect in the current meta-analysis, because the ROC curve was not a typical ‘shoulder-arm’ pattern (Figure 3). The Spearman correlation coefficient between the logarithm of sensitivity and the logarithm of 1-specificity was 0.6 (*P*=0.285) so the difference was not statistically significant. Overall, the diagnostic accuracy of miR-126 for NSCLC was as follows: pooled sensitivity = 0.83 (95% CI: 0.59–0.94), specificity = 0.83 (95% CI: 0.71–0.90), PLR = 4.78 (95% CI: 2.97–7.69), NLR = 0.20 (95% CI: 0.08–0.54), DOR = 23.48 (95% CI: 7.87–70.10), AUC = 0.89 (95% CI = 0.86–0.91). The forest plot of DOR, the sensitivity and specificity of forest plot and the forest plots of PLR and NLR were respectively shown in Figure 4A–C. The SROC curve was shown in Figure 3, AUC = 0.98 (95% CI: 0.96–0.99). Fagan’s Nomogram result showed that the pre-test probability ratio was 20%. The post-test probability of PLR was 54%, NLR was 5% (Figure 4D). It indicated that miR-126 had a good diagnostic performance for NSCLC. The Deeks funnel plot in Figure 5 showed that *P*-value was 0.61, which indicated that it is almostly equal to no publication bias.

**Figure 6:**
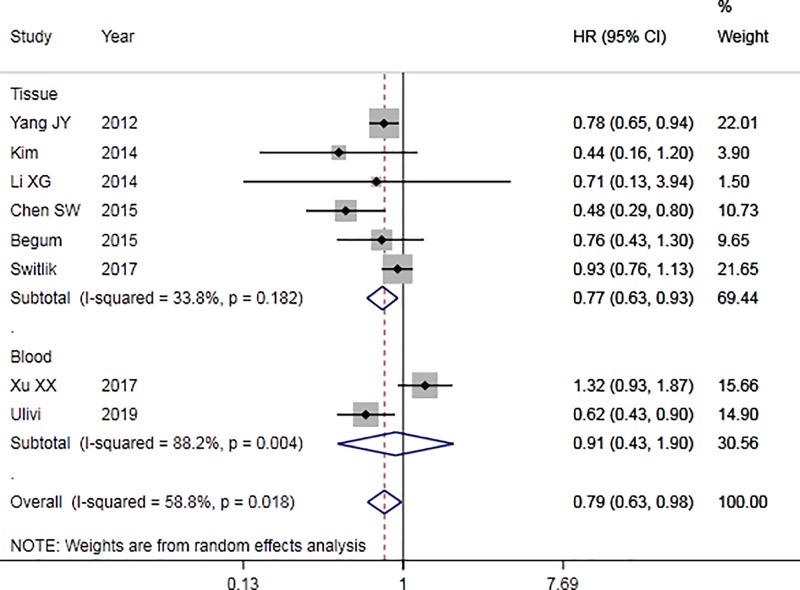
Forest plot of miR-126 for the prognosis (OS) of NSCLC

**Figure 7:**
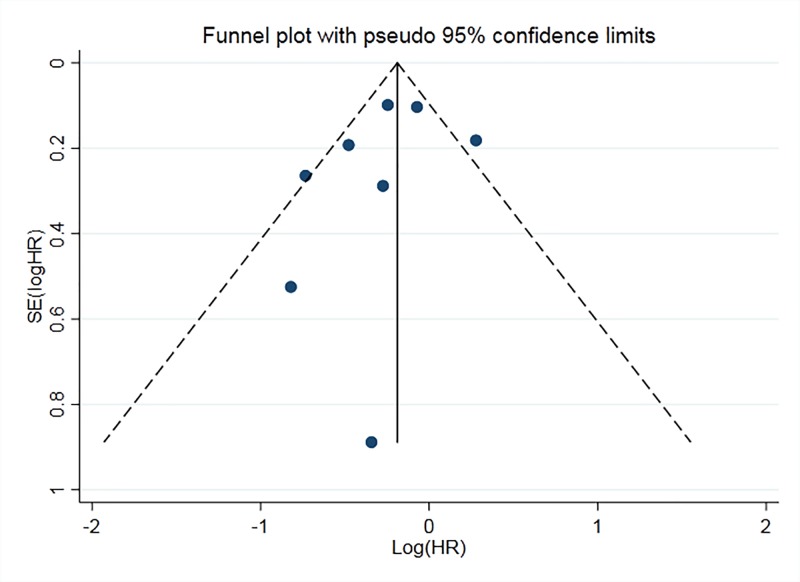
Funnel plot of miR-126 for the prognosis (OS) of NSCLC Abbreviation: SE, standard error.

To assess the association between miR-126 expression and OS in NSCLC, the forest plot and meta-analysis of individual HR estimates was shown in Figure 6, and the results of subgroup analysis was shown in Table 3. According to Figure 6, *I^2^* = 58.8% (*P*<0.05), which indicated that there was heterogeneity between studies. Random-effects model was used to calculate the combined HR and its 95% CI. Comparison between high expression and low expression of miR-126, the result of meta-analysis showed that the pooled HR was 0.79 (95% CI: 0.63–0.98). The results of ethnic subgroup analysis showed that the heterogeneity did not decrease. The subgroup analysis of the sample source showed that the heterogeneity decreased significantly with *I^2^* = 33.8% (*P*<0.05) in the subgroup of the tissue specimen, indicating that the sample source was the main source of heterogeneity. The results showed that the difference of OR = 0.77 (95% CI: 0.63–0.93) was statistically significant in tissue subgroup. The funnel plot (Figure 7) was basically symmetrical, and Egger’s test showed that the difference was not statistically significant (*P*>0.05), indicating that there was no publication bias. These results suggest that there was significant difference in OS between high expression of miR-126 and low expression of NSCLC.

**Table 3:** Results of subgroup analysis of prognostic value

Prognosis	Subgroup	*n*	HR	95% CI	*P*-value	*I^2^* (%)	*P* for heterogeneity	Model	*P* for publication bias
Overall		8	0.79	0.63–0.98	0.029	58.8	0.018	REM	0.383
Specimen	tissue	6	0.77	0.63–0.93	0.008	33.8	0.182	FEM	0.193
	Blood	2	0.91	0.43–1.90	0.796	88.2	0.004	REM	NA
Ethnicity	Asian	5	0.76	0.51–1.13	0.176	69.7	0.01	REM	0.685
	Caucasian	3	0.79	0.60–1.04	0.094	46.5	0.155	REM	0.467

Abbreviations: FEM, fixed-effect model; REM, random-effect model.

### Sensitivity analysis

**Figure 8:**
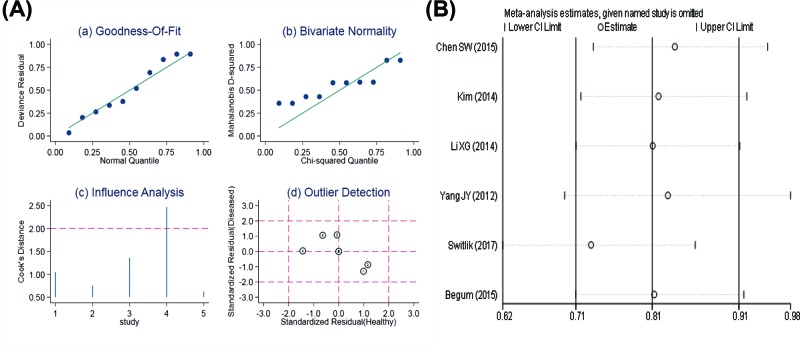
The results of sensitivity analysis (**A**) Diagnostic value; (**B**) prognostic value.

The sensitivity analysis of the diagnostic value was shown in Figure 8A. Goodness-of-fit and bivariate normal analysis showed that the bivariate random-effect model was robust for meta-analysis. In addition, one bias study that may affect the robustness of meta-analysis was identified through influence analysis and outlier detection. After the exclusion study, no significant changes in sensitivity (0.83 vs. 0.74), specificity (0.83 vs. 0.81), PLR (4.78 vs. 3.90), NLR (0.20 vs. 0.32), DOR (23.48 vs. 12.00), and AUC (0.89 vs. 0.85) were observed between the overall analysis with and without outlier, suggesting that the meta-analysis of diagnostic value in the present study was highly robust.

The sensitivity analysis of the prognosis of miR-126 and NSCLC was shown in Figure 8B. Due to the low heterogeneity of the results for tissue sources, we specifically analyzed the sensitive results of this subgroup. After the included literatures were excluded one by one, meta-analysis was performed again to observe the impact of each study on the OS rate. The results showed that the change in HR was not statistically significant after excluding the literatures one by one. This suggests that the included studies can highly support the results. In other words, for patients with NSCLC, the prognosis of high expression of miR-126 is better than that of low expression of NSCLC.

## Discussion

miR-126 has aroused great interest as a new biomarker for diagnosis and treatment of NSCLC. The expression of miR-126 in NSCLC was significantly different from that of healthy controls [31]. miR-126 plays an extensive role in NSCLC. For example, at the cellular level, it has been found that miR-126-3P inhibits the growth, migration, and invasion of NSCLC by targeting CCR1 in NSCLC cells [32]. The expression of miR-126 can be enhanced by negatively regulating the VEGF/PI3K/Akt/MRP1 signaling pathway, which can increase the sensitivity of NSCLC cells to anticancer drugs [33]. Moreover, miR-126 is involved in regulating the response of NSCLC cells to cancer therapy. For example, miR-126 can promote radiation-induced NSCLC cell apoptosis through the PI3K-Akt pathway [34]. In addition, the studies conducted by Shen et al. [35] have shown that the sensitivity and specificity of combining miR-21, miR-126, miR-210, and miR-486-5p in distinguishing between NSCLC patients and healthy controls are as high as 86.22 and 96.55%. These studies show that miR-126 plays a key role in the biology of NSCLC cells, which also indicate that it is a promising target for gene diagnosis and therapy for patients with NSCLC. The differential diagnostic value and prognostic value of miR-126 in patients with NSCLC have been found by previous researchers, but their conclusions are inconsistent. Moreover, meta-analysis of NSCLC in the past are rarely reported. So in the present study, after obtaining a large sample size and integrating individual data with appropriate methods, a meta-analysis was conducted to evaluate the role of miR-126 in the diagnosis and treatment of NSCLC.

In the comprehensive analysis of diagnostic accuracy of miR-126 and NSCLC, five studies were included, including 493 patients with NSCLC and 463 healthy people. The results showed that the combined sensitivity was 0.83 and combined specificity was 0.83, PLR = 4.78, NLR = 0.20, DOR = 23.48 (95% CI: 7.87–70.10), and the area under of SROC curve was 0.89 (95% CI: 0.86–0.91). From the Spearman correlation test of logarithm of sensitivity and logarithm of 1-specificity, as well as the shape of the SROC curve, there was no significant threshold effect between the included studies. From the sensitivity, specificity and the area under the SROC curve, miR-126 had diagnostic value for NSCLC. But combined with PLR and NLR, PLR was less than 10 and NLR was greater than 0.1, so the ability of miR-126 in diagnosing NSCLC was still limited. Therefore, in practice, the diagnostic accuracy of NSCLC can be improved by combining miR-126 with other biomarkers to diagnose NSCLC.

In the comprehensive analysis of the prognostic value of miR-126 for NSCLC, a total of eight studies were included, including 1102 patients. The results showed that in the OS risk analysis of miR-126 and NSCLC, combined HR = 0.79 (95% CI: 0.63–0.98), so the difference was statistically significant. Because there was heterogeneity in the study, the random-effect model was used to analyze. The results of ethnic subgroup analysis showed that there was no significant decrease in heterogeneity in Asian and Caucasian populations, so ethnicity may not be the main source of heterogeneity. The results of the sample source showed that the subgroup heterogeneity of the sample from the tissue decreased significantly, which supported the correlation between the expression of miR-126 and NSCLC, and the sensitivity analysis to it further supported this conclusion. From the results of publication bias, the *P*-value of Egger’s test was more than 0.05, and funnel plot was basically symmetrical, so it could be considered that there was no publication bias. From the sensitivity analysis, after removing the single study and re-meta-analysis, there was no statistically significant change in HR. For the time being, it could be considered that there was a correlation between the expression of miR-126 and the OS of patients with NSCLC. In a meta-analysis of four studies conducted by Zheng et al. [36] shows that the high expression of miR-126 was a favorable factor for OS in patients with NSCLC. It was consistent with our conclusion, and more importantly, more high-quality literatures were included to support this view in our research.

However, the present study also had some limitations: (1) in terms of the diagnostic accuracy, only five studies met the criteria of combined analysis, and the samples were taken from plasma, serum and sputum, which may have a certain effect on the heterogeneity; (2) there was a great heterogeneity in the accuracy of miR-126 in the diagnosis of NSCLC. The source of this heterogeneity may be due to the tumor stage, the source of samples etc, but due to the limited number of studies, it was impossible to perform subgroup analysis to determine the source of heterogeneity; (3) there may be a certain correlation between miR-126 and chemotherapy sensitivity, which may also affect the prognosis of patients; (4) the present study only focused on the meta-analysis of diagnostic value and prognostic value of miR-126 in NSCLC, not combined with other possible biomarkers.

In conclusion, miR-126 is a promising biomarker for the diagnosis and prognosis of NSCLC, with high sensitivity and specificity, and high expression of miR-126 has a better prognosis than low expression. It can also combine with other biomarkers, such as miR-21 and miR-210, to diagnose NSCLC patients. It provides a faster and less invasive assessment toward NSCLC patients than other markers that require histopathological analysis. Our results gave evidence for the valuable diagnostic role of miR-126 in NSCLC, which may ultimately contribute to the understanding of the role of miR-126 in the early diagnosis of NSCLC. At the same time, miR-126 shows a prognostic value for NSCLC, which is good news for lung cancer patients, and targeted treatment will achieve better efficacy. Considering that there were still some limitations in the present study, more high-quality studies are needed to explore the diagnostic and prognostic value of miR-126 in patients with NSCLC.

## Conclusions

Our results give evidence for the valuable diagnostic role of miR-126 in NSCLC, which may ultimately contribute to the understanding of the role of miR-126 in the early diagnosis of NSCLC. Compared with tissues, the markers in blood or secretion have more practical value in the diagnosis of NSCLC, for they are easy to obtain and have less damage to the body. Of course, in practical application, miR-126 can be combined with patients’ clinical manifestations, laboratory tests, or other biomarkers to diagnose NSCLC to improve its diagnostic accuracy. The study do support the effect of miR-126 on the prognosis of patients with NSCLC. For patients with NSCLC, the prognosis of high expression of miR-126 is better than that of low expression. This result is positive for the treatment and prognosis of patients with NSCLC, and it can be said to be of great significance. Considering that there were still some limitations in the present study, more high-quality studies are needed to explore the diagnostic and prognostic value of miR-126 in patients with NSCLC.

## Abbreviations

AUC: area under the curve
CI: confidence interval
DOR: diagnostic odds ratio
HR: hazard ratio
*I^2^*: inconsistency index
miR-126: microRNA-126
NLR: negative likelihood ratio
NOS: Newcastle–Ottawa scale
NSCLC: non-small cell lung cancer
OS: overall survival
PLR: positive likelihood ratio
QUADAS: quality assessment of diagnostic accuracy studies
SCLC: small cell lung cancer
SROC: summary receiver operating characteristic curve
